# Economic evaluation of advanced practice physiotherapy models of care for upper extremity musculoskeletal disorders In Denmark: a registry-based cohort study

**DOI:** 10.1007/s10198-025-01817-z

**Published:** 2025-08-04

**Authors:** Simon Lafrance, Cecilie Rud Budtz, Martin Byskov Kinnerup, François Desmeules, Jason Robert Guertin, Merete Nørgaard Madsen, David Høyrup Christiansen

**Affiliations:** 1 Department of Social and Preventive Medicine, Université Laval, Quebec City, QC Canada; 2 Centre de recherche du CHU de Québec – Université Laval, Quebec City, QC Canada; 3 Silkeborg Regional Hospital, University Clinic for Interdisciplinary Orthopaedic Pathways (UCOP), Falkevej 3, 8600 Silkeborg, Denmark; 4 School of Rehabilitation, Faculty of Medicine, Université de Montréal, Montreal, QC Canada; 5 Maisonneuve-Rosemont Hospital Research Center, Université de Montréal Affiliated Research Center, Montreal, QC Canada; 6 Centre de Recherche en Organogénèse Expérimentale de L′Université Laval/LOEX, Quebec City, QC Canada; 7 Department of Clinical Medicine, HEALTH, Aarhus University, Aarhus, Denmark; 8 Centre for Research in Health and Nursing, Research, Regional Hospital Central Jutland, Viborg, Denmark

**Keywords:** Advanced practice, Physiotherapy, Upper extremity, Shoulder, Health care organization, Health economic, Denmark

## Abstract

**Background:**

Advanced practice physiotherapy (APP) models of care, which provide greater autonomy and responsibility to physiotherapists, have emerged as promising solutions to increase healthcare access while providing cost-effective care for MSK disorders. A formal health economic evaluation of these models has yet to be undertaken in Denmark.

**Objectives:**

To perform a registry-based economic evaluation of APP care versus standard care models for managing upper extremity MSK disorders in four Danish orthopedic clinics in the societal perspective.

**Methods:**

Data related to sociodemographic, diagnoses, healthcare resource use, medication, costs, and sickness benefits within a two-year period after the initial consultation were extracted from Danish databases. Total healthcare costs including primary care (medical and rehabilitation), medication and hospital costs were calculated as well as productivity loss. Costs were converted to Euros 2022. Propensity score weighting was used to adjust for confounders.

**Results:**

A total of 13,517 patients were included in the main analysis. Healthcare cost distribution differed between the two models with higher rehabilitation (mean difference [MD]: €18; 95% CI: 8 to 28) but lower medication (MD: -€50; 95% CI: -58 to -43) costs with the APP model of care. However, there was no significant difference in total healthcare costs (MD: €86; 95% CI: -305 to 476) nor in productivity loss (MD: €197; 95% CI: -1678 to 2072) between the two models.

**Conclusion:**

APP care results in similar total healthcare costs and productivity loss when compared to standard care for adults with upper extremity MSK disorders.

**Supplementary Information:**

The online version contains supplementary material available at 10.1007/s10198-025-01817-z.

## Background

The rising prevalence of musculoskeletal (MSK) disorders poses substantial challenges to global healthcare systems, which are already strained due to limited resources [1–4]. In this context, optimizing healthcare delivery has become essential for managing MSK disorders. Meanwhile, the share of gross domestic product allocated to healthcare in OECD countries has nearly doubled over the past five decades [5]. Considering the rising demand for MSK care and the constant increase in healthcare cost [4, 6, 7], governments and healthcare systems need to find cost-effective solutions to manage MSK disorders. This is notably the case in Denmark, where the economic burden related to MSK disorders is substantial. A recent nationwide study estimated that shoulder disorders alone generate over €1.2 billion in annual societal costs, primarily driven by productivity losses associated with a high prevalence of 1,215 cases per 100,000 person-years [7]. This highlights the importance of developing sustainable and efficient care models to deliver cost-effective care for individuals with MSK disorders.

Given these challenges, advanced practice physiotherapy (APP) models of care, which are physiotherapist-led models, have emerged as promising solutions [8, 9]. In these models, physiotherapists are granted greater autonomy and responsibility, enhancing their roles and allowing them to perform tasks traditionally reserved to medical doctors [10, 11]. Previous systematic reviews reported that APP models of care improve healthcare access by reducing waiting times for initial consultations and for referrals to medical specialists when necessary [12–15]. These models also provide care that is comparable, or even superior, to standard models in terms of pain and disability reduction [16]. Additionally, APP models are associated with lower healthcare costs per patient, primarily due to reduced surgical referral rates, lower salaries, and decreased medication prescriptions [17]. This was notably the case in an orthopedic study conducted in the United Kingdom, where patients managed under an APP model were 2.4 times less likely to be placed on a surgical waiting list, resulting in a cost reduction of €473 per patient [17, 18].

Within the Danish healthcare system, some hospitals have integrated these models, where advanced practice physiotherapists (APPTs) assume greater responsibility [19, 20], while many others persist with the physician-led standard medical model of care. Although international studies suggest that broader implementation of APP models of care may lead to significant cost savings while maintaining or even improving clinical outcomes [16, 17], these findings are context-dependent. This is because economic evaluations are influenced by local factors such as healthcare financing, provider salaries, referral pathways, and access to surgical care, which limits the generalizability of findings across different settings or countries. Therefore, a formal health economic evaluation of APP models within the Danish healthcare system is essential to determine their value, especially considering the need for cost-effective care models, and the substantial economic burden posed by shoulder MSK conditions in Denmark [7, 17].

Therefore, our aim is to perform an economic evaluation comparing APP models of care with the standard medical model for the management of adults with upper extremity MSK disorders in orthopedic clinics in Denmark, using national registry-based data.

## Methods

### Study design and ethics

This is a registry-based observational longitudinal study from the societal perspective. The RECORD and CHEERS checklists are reported in supplementary material [21, 22]. The protocol was written a priori but was not published. According to Danish law, registry-based studies are not required to obtain permission from the regional ethics committee.

### The danish healthcare system

The Danish healthcare system is considered a universal healthcare system and is divided into five regions, including Central Denmark. The Danish population has free access to most healthcare services, which are primarily tax-financed [23]. Some healthcare services, such as physiotherapy care, are only partly financed by the public healthcare system and additional costs are covered by private insurances or patient out-of-pocket expenses.

### Study setting and models of care

The APP model of care established at the shoulder and upper extremity clinics in Silkeborg and Viborg regional hospitals (APP model of care) will be evaluated, while the standard medical model of care at two shoulder clinics in Horsens and Randers regional hospitals in Central Denmark will serve as the comparator (standard model of care). Patients at the Silkeborg or Viborg shoulder and upper extremity clinics are assessed and managed by APPTs and/or orthopedic surgeons working collaboratively. Depending on the conditions and needs of the patients, the APPT, the surgeon, or both may be involved in their care. For example, the APPT may perform an independent assessment but will consult the orthopedic surgeon if the treatment plan includes surgery, a corticosteroid injection, a referral for advanced diagnostic imaging, or if a more comprehensive medical evaluation is required. The standard medical model of care, defined as an orthopedic surgeon-led model with minimal or no involvement of physiotherapists, was practiced at the Randers and Horsens shoulder and upper extremity clinics during the inclusion period.

The Silkeborg Regional Hospital is in the Silkeborg Municipality, which has 97,400 citizens, and the Viborg Regional Hospital is in the Viborg Municipality, which has 97,731 citizens. The two comparators are located in Randers Municipality, which has 99,931 citizens, and Horsens Municipality, which has 96,480 citizens [24]. The four clinics are located in the Central Denmark Region and we assumed that similar population are consulting in these clinics.

### Population and eligibility criterias

All patients from Central Denmark Region were identified based on data from the Danish National Patient Register. To be included, patients needed to 1) be at least 18 years old; 2) have consulted in one of the participating clinics for a new upper extremity MSK disorders between 2016/01/01 and 2017/12/31; and 3) have received a primary diagnosis related to an upper extremity MSK disorders including: shoulder disorders (ICD-10: M75*, excluding M750), frozen shoulder (ICD-10: M750), injury or sequela of the upper extremity (ICD-10: T92* and S4*), osteoarthritis (ICD-10: M19*), luxation or subluxation (ICD-10: M244*) and other (ICD-10: M253, M357, Z966C, and G54* but excluding G542 and G544). Patients were excluded if, during the 2-year period following the initial consultation, they 1) received a diagnosis of malignant neoplasms (ICD-10: C00-97); 2) emigrate outside of Denmark; or 3) die. The rationale for excluding these patients was that neoplasms or death would lead to high healthcare costs unrelated to upper extremity MSKD, and the Danish database does not capture healthcare costs occurring outside of Denmark.

### Registries and data extraction

In Denmark, the Danish Civil Personal Register number, a unique identifier for each resident, links information about all health and social events from birth to death. Using this number, patients’ data, such as healthcare resources use, were linked at the individual level. We used the CROSS-TRACKS population-based open cohort of individuals resident in Central Denmark Region [25], to link:

- patients'sociodemographic data (e.g., age, gender, vital status, emigration) from the Danish Civil Registration System [26];
- primary care contact and costs charged to the healthcare system (e.g., physician visits, other medical costs, private physiotherapy visits, private chiropractor visits) from the Danish National Health Services Register [27];
- medication prescriptions (types, number of prescriptions, and number of units per prescription) from the Prescription Registers and electronic patient journals [28];
- referral to public physiotherapy rehabilitation from electronic patient journals;
- information on diagnosis, hospital inpatient and outpatient visits from the Danish National Patient Register [29, 30]; and
- sickness benefit data and employment status from the DREAM register [31].

The Charlson Comorbidity Index was calculated based on healthcare data from 10 years prior to the initial consultation date from the Danish National Patient Register [29, 30]. Data related to sociodemographic information, other diagnoses, healthcare resources and services use, medication, costs, and sickness benefits within a two-year period after the initial consultation (from 2018/01/01 to 2019/12/31, depending on the initial appointment date) were extracted.

**Fig. 1:**
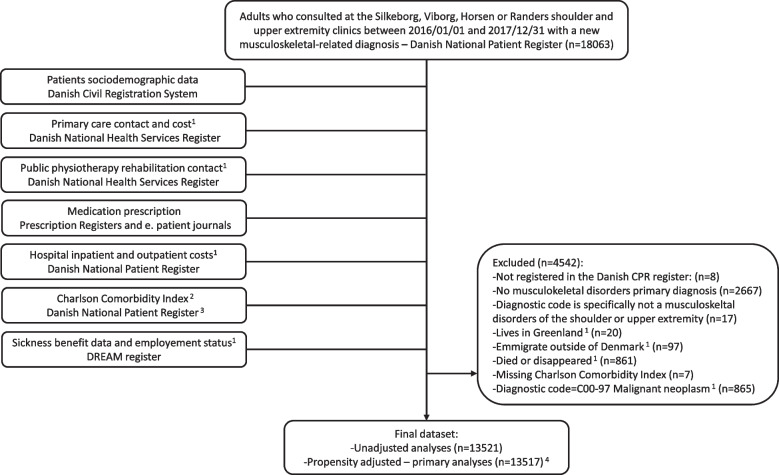
Flowchart and data linkage

The linkage of database is also presented in Fig. 1. We used previously validated codes and algorithms to select the population and data [32–39]. Data were extracted using the Stata software (StataCorp. 2023. Stata Statistical Software: Release 18. College Station, TX: StataCorp LLC).

### Costing method

Primary care costs were directly extracted from the database as the costs charged by medical clinics, physiotherapy clinics, or chiropractic clinics to the healthcare system. The cost of physiotherapy and chiropractic care from the private sector is reimbursed at 40% and 18 to 20% by the state, respectively [40, 41]. The cost for physiotherapy rehabilitation in the public system has been previously estimated at 365 DKK (€49.06) per 1-h visit in 2018 and the mean number of consultations being 9.7 per referral [42]. Which was used to estimate the cost of rehabilitation. Since we did not have access to the proportion of individuals who used the service, we assumed that 80% of individuals with a prescription for physiotherapy rehabilitation in the public system would have utilized the service. This estimate was informed by the expertise of Danish researchers on our team. The cost of medication was estimated based on the average price per unit for each medication code (ATC codes) as provided by the Danish Medicines Agency [43]. The cost of each prescription was calculated as the number of units prescribed multiplied by the cost per unit for a given medication. For medications administered at the hospital, we assumed that one unit per day was prescribed. Hospital inpatient and outpatient costs were directly extracted from the Danish National Patient Register, with the total hospital cost (including all hospital costs encountered during the period regardless of the disorder) and upper extremity hospital costs, which include all hospital cost related to episodes of care for an upper extremity disorders based on the ICD-10 codes (M75*, T92*, S4*, M19*, M244*, M253, M357, Z966C and G54* but excluding G542 and G544), with the exception of medication costs. Productivity loss was calculated based on the mean yearly salary in the Central Denmark Region in 2022, which was 519,384 DKK (€69,810) per year [44], and the number of weeks on sick leave per patient using the human-capital method [45].

Costs were adjusted for inflation based on the Danish consumer price index, except for medication costs as the current 2022 cost per unit was extracted [43]. For patients with an initial consultation in 2016, the mean consumer price index for 2016–2018 was calculated and adjusted to 2022 (coefficient = 1.12). The same procedure was performed for patients with an initial consultation in 2017, using the mean consumer price index for 2017–2019 (coefficient = 1.1099) [46]. Costs were then converted to Euros (7.44 DKK = €1) [47].

### Statistical analyses

Descriptive statistics, including age, sex, Charlson comorbidity index, civil status, and diagnosis, were used to describe the cohort. Healthcare resource use including the number of visits to family physicians, medical specialists, physical therapists, and chiropractors in primary care; the number of hospital admissions; the number of days of admission; and the number of ambulatory consultations at the hospital were presented per models of care and clinics. Means and standard deviations were calculated for continuous data, and numbers and proportions were calculated for categorical data.

To adjust for potential confounders, a propensity score was calculated using a multivariate logistic regression model that included the following covariates: age, sex, and Charlson comorbidity index. Due to the uncertain validity of the diagnosis data, which is not used for reimbursement purposes, diagnoses were not included in the regression model. A propensity score was assigned to each individual. The propensity score distribution was analyzed, and patients with a propensity score in a non-overlapping area were excluded from the analysis. For the primary analysis, propensity score weighting was performed, where each individual was weighted by the inverse of their propensity score [48].

Estimated costs: primary care (medical and rehabilitation), medications, hospital (total and upper extremity-related) and total healthcare costs (primary care + medications + total hospital costs) as well as productivity loss per patients in both groups were calculated as well as the between-group differences.

Alpha levels were fixed at 0.05 for all statistical tests. All statistical analyses were performed using the R Statistical Software (version 4.3.1).

## Results

### Data linkage and extraction

This cohort study initially considered 18,063 adults registered who consulted for a new upper extremity disorder at the Silkeborg, Viborg, Horsens, or Randers clinics from January 1, 2016, to December 31, 2017. In total, 4,542 patients were excluded. The final dataset includes 13,521 patients for unadjusted analyses and 13,517 patients for propensity adjusted analyses, as presented in Fig. 1.

### Cohort description

Participants cared in the APP and in standard care clinics had similar baseline characteristics overall. Mean age was 50.8 ± 15.6 years old, 50.1% were female, most were married (58.7%) and were diagnosed with various upper extremity disorders, mainly involving the shoulder. Patients’ characteristics per model of care are presented in Table 1 and in per clinic in Table 4 of the supplementary material.

**Table 1:** Cohort characteristics per group

	**Standard model of care**	**APP model of care**
Cohort, n	7561	5960
Age, mean ± SD	50.9 ± 15.8	50.8 ± 15.5
Female, %	49.1%	51.3%
Civil status, n (%)		
-Married^1^	4349 (57.5%)	3606 (60.5%)
-Divorced or widowed^2^	1426 (18.9%)	1058 (17.8%)
-Not married	1735 (22.9%)	1263 (21.2%)
-Unknown	51 (0.7%)	33 (0.6%)
Charlson Index, mean ± SD	0.28 ± 0.74	0.29 ± 0.76
Upper extremity diagnoses, n (%)		
-Shoulder disorders^3^	3890 (51.4%)	3558 (59.7%)
-Frozen shoulder	451 (6.0%)	504 (8.5%)
-Injury or sequela of UE^4^	153 (2.0%)	124 (2.1%)
-Osteoarthritis^4^	1940 (25.7%)	830 (13.9%)
-Luxation or subluxation^4^	1105 (14.6%)	912 (15.3%)
-Other	22 (0.3%)	32 (0.5%)

*APP* Advanced practice physiotherapy; *SD*: Standard deviation; *UE*: Upper extremity

^1^Include registered partnership

^2^Include dissolved partnership

^3^Excluding frozen shoulder

^4^Not joint-specific

### Healthcare cost

The number of visits in primary care as well as hospital admission and ambulatory visits were similar in both groups as presented in Table 2.

**Table 2:** Care received per clinic and per group

	**Standard model of care**	**APP model of care**
Participants, n	7561	5960
Visits, mean ± SD		
-Physician	21.13 ± 18.82	21.56 ± 18.91
-Physiotherapist	4.07 ± 11.01	3.65 ± 10.81
-Chiropractor	0.45 ± 2.41	0.38 ± 2.32
-Orthopedic surgeon	0.05 ± 0.45	0.03 ± 0.35
Hospital, mean ± SD		
-Admission number	0.28 ± 1.1	0.28 ± 1.13
-Admission days	0.78 ± 2.13	0.78 ± 2.08
-Ambulatory visit	2.07 ± 9.1	2.19 ± 11.52

*APP* Advanced practice physiotherapy; *SD* Standard deviation; *UE* Upper extremity

Primary care medical costs were similar in both models of care (MD: €0; 95% CI: −21 to 21), while rehabilitation cost was higher with APP model of care (MD: €18; 95% CI: 8 to 28). Among the whole cohort, the mean primary care cost was €546/patient (Table 3).

**Table 3:** Propensity score adjusted mean healthcare cost and productivity loss per group and between-group differences per patients

	**Standard model of care; n = 7560**	**APP model of care; n = 5964**	**Between-group difference**
	**Mean € (95% CI)**	**Mean € (95% CI)**	**Mean € (95% CI)**^**1**^
Primary care medical	444 (434 to 455)	444 (433 to 454)	0 (−21 to 21)
Primary care rehabilitation	94 (89 to 98)	112 (106 to 118)	18 (8 to 28)
Medications	65 (59 to 70)	15 (12 to 17)	−50 (−58 to −43)
Hospital total	3219 (3037 to 3401)	3305 (3096 to 3513)	86 (−305 to 476)
*Hospital upper extremity*^*2*^	*1160 (1129 to 1191)*	*1090 (1059 to 1122)*	*−69 (−132 to −7)*
Total healthcare cost^3^	3822 (3636 to 4007)	3875 (3662 to 4087)	53 (−345 to 451)
Productivity loss	15,814 (14,926 to 16,701)	16,011 (15,023 to 16,998)	197 (−1678 to 2072)

*APP* Advanced practice physiotherapy; *CI* Confidence interval; *MSK* Musculoskeletal

^1^Positive between-group difference indicates higher costs per patients in the APP model of care

^2^Hospital upper extremity include cost related to episodes of care for an upper extremity disorders based on the ICD-10 codes

^3^Total healthcare costs = Primary care medical + Primary care rehabilitation + Medications + Hospital total

Medication costs were lower with APP care (MD: -€50; 95% CI: −58 to −43). Among the whole cohort, the mean medication cost was €43/patient.

The difference in hospital costs was not statistically significant (MD: €86; 95% CI: −305 to 476) except for upper extremity-related costs (MD: -€69; 95% CI: −132 to −7), which were lower with the APP model of care. Among the whole cohort, the mean hospital cost was €3,257/patient, from which €1,129 were specific to upper extremity care.

The difference in total healthcare costs, including primary care, medication, and hospital costs, was not statistically significant (MD: €53; 95% CI: −345 to 451). Among the whole cohort, the mean total healthcare cost was €3,845/patient.

Similar results were obtained from unadjusted and per clinic analyses as presented in supplementary materials (Tables 6–8).

### Productivity loss

The difference in productivity loss was not statistically significant (MD: €197; 95% CI: −1678 to 2072). There were 2,223 (29.4%) and 1,776 (29.8%) patients had productivity loss among the standard and APP care models respectively. Among the whole cohort, the mean productivity loss was €15,900/patient (Table 3).

## Discussion

### Main findings

Overall, there was no significant difference in total healthcare costs nor productivity loss for adults consulting for a new upper extremity MSK disorders in an APP care model or in the standard care model in Central Denmark. However, small but statistically significant between-group differences were observed in the cost of primary care rehabilitation, medication, and upper extremity-related hospital costs. Specifically, costs for medication and upper extremity-related hospital costs were lower in the APP care model, while the rehabilitation costs were higher.

### Strength and limitations

This study includes a large cohort with an extensive dataset, covering a wide range of descriptive data, diagnoses, and costs. Regarding data validity, primary care and hospital costs are considered highly reliable, as these administrative data are used for reimbursement in Denmark. However, some assumptions were made about medication use and public physiotherapy rehabilitation, as we only had access to prescription data. The validity of the diagnostic data remains uncertain, and we cannot exclude the possibility of variations in diagnostic coding between clinics (e.g., shoulder osteoarthritis might be coded under both shoulder disorders and osteoarthritis). Consequently, diagnostic information was not included in the propensity score. Included data extends beyond the healthcare system to include productivity losses, offering a societal perspective of the economic impact. However, costs from the patient’s perspective were not included as they are not present in Danish registries, limiting the societal perspective to healthcare cost and productivity losses. Private healthcare costs (hospital and rehabilitation) were not included as they are not present in Danish registries, which led to an underestimation of the total healthcare cost. We had to make an assumption regarding the proportion of individuals who received public physiotherapy rehabilitation, based on the number of referrals, and estimated that 80% sought these services. The between-group difference in public physiotherapy rehabilitation costs was relatively small at €28 per patient, with higher costs in the APP group. This assumption had minimal impact on our results, as varying the proportion to 95% or 65% led to differences of €33 and €23, respectively. These variations would not have altered our conclusions regarding rehabilitation or total healthcare costs. Regarding the healthcare cost, it was not possible to extract only cost specific to the initial MSK condition; therefore, we had to compare total healthcare cost which includes consultation unrelated to the upper extremity MSK disorder. However, we assumed that costs unrelated to the initial condition would be distributed similarly between groups, allowing for valid comparative analysis between the two models. Furthermore, upper extremity-related hospital costs were analyzed.

### Interpretation and clinical implications of the results

In our study, patients seen in an APP model of care for an upper extremity MSK disorders led to higher rehabilitation cost, but lower medication cost. This suggests that the care management in these two models was different, with patients in the APP care setting used more rehabilitation services but less medication than those cared in a traditional orthopedic setting. This is not surprising, as patients in the physiotherapy-led model received more rehabilitation, an essential component of physiotherapy care, and fewer medication prescriptions, which are more typically used by physicians. However, these differences were relatively small and did not significantly impact the total healthcare costs. Hospital upper extremity-related costs were also lower with APP care. However, uncertainty remains as this was not observed in total hospital costs, where the between-group difference was not significant. The large confidence intervals, driven by a small number of patients with very high costs, as commonly observed in economic data, highlight the imprecision of our results due to important individual cost variability despite our large sample size.

The current literature, including our findings, suggests that APP models of care are associated with comparable or even lower healthcare costs than standard models of care, although economic data remain context-dependent. Given that APP models result in similar or superior clinical outcomes in terms of pain, disability, and health-related quality of life, they are likely to be more cost-effective than standard care. In some cases, they may even be considered dominant, delivering better outcomes at a lower cost.

As often observed, the variability in productivity loss was substantial, with a minority of patients driving this productivity loss due to prolonged work absenteeism. This aspect should be further studied as the current results are too imprecise to draw definitive conclusions. It is also possible that other determinants, such as psychosocial and work-related factors, play a more significant role in productivity loss than the model of care itself.

### Comparison with the literature

A previous meta-analysis estimated that APP care led to a mean healthcare cost reduction of €139/patient when compared to standard care for MSK disorders [17]. This reduction was notably driven by a substantial €473 per patient cost reduction in an orthopedic study, as fewer patients were placed on surgical waiting lists [18]. In contrast, our study found a more modest reduction in upper extremity-related hospital costs of €69 per patient in the APP model. Although our results on total healthcare cost reduction did not reach statistical significance, the confidence interval is wide and overlapped with the one from this meta-analysis. Although this systematic review included studies conducted in Western countries, none were conducted in Denmark and only one was conducted in a Scandinavian country, in occurrence Sweden. This aspect must be underlined in the comparison with the literature as economic evaluations are highly context-dependent and can differ between two clinical settings or countries. Productivity loss estimates were highly imprecise in both the meta-analysis and in our study, highlighting the presence of substantial variability. There is a need for high-quality research, which also assesses the impact of other determinants, such as psychosocial and work-related factors, on productivity losses [17].

### Unanswered questions and future research

Uncertainty remains, notably due to the nature of our study design using registry-based data. Future studies should include prospective enrollment of patients and data collection performed for research purposes.

Productivity loss should be further studied, due to the large variability and imprecision of productivity loss. Prospective studies documenting in more detail on patient characteristics, job demands, and contributing factors to productivity loss could enable more nuanced and stratified analyses. Such data would allow for a deeper understanding of the impact of different models of care and interventions on work-related disability and productivity loss.

## Conclusion

In this Danish registry-based cohort of adults consulting for an upper extremity MSK disorders in an APP or traditional orthopedic clinic. The distribution of health care cost slightly differs with higher rehabilitation and lower medication cost in the APP model. Upper extremity-related hospital costs were slightly lower in the APP model; however, total hospital costs did not significantly differ between groups. Overall, there was no statistically significant difference in total healthcare costs over a 2-year period. Similarly, there was no significant between-group difference in productivity loss over a 2-year period. Large confidence intervals, for health care cost and especially for productivity loss limit the certainty of our conclusions. These results are influenced by the wide range of factors affecting overall healthcare costs. Future studies should investigate the impact of various factors on both healthcare costs and productivity losses.

## Supplementary Information

Below is the link to the electronic supplementary material.

Supplementary file1 (DOCX 45 KB)

## Data Availability

Study data are available from the corresponding author, upon reasonable request and according to the Danish Law.

## Abbreviations

APP: Advanced Practice Physiotherapy
APPT: Advanced Practice Physiotherapists
ATC: Anatomical Therapeutic Chemical (classification system)
CHEERS: Consolidated Health Economic Evaluation Reporting Standards
CI: Confidence Interval
DKK: Danish Krone
DREAM: Danish Register-based Evaluation of Marginalization
GDP: Gross Domestic Product
ICD-10: International Classification of Diseases, Tenth Revision
MD: Mean Difference
MSK: Musculoskeletal
OECD: Organisation for Economic Co-operation and Development
RECORD: REporting of studies Conducted using Observational Routinely collected health Data
