# Supplementary material for: Economic evaluation of advanced practice physiotherapy models of care for upper extremity musculoskeletal disorders In Denmark: a registry-based cohort study
Source: Eur J Health Econ. 2025 Aug 4;27(2):363–72. doi: 10.1007/s10198-025-01817-z (PMC13046617; doi:10.1007/s10198-025-01817-z)
Supplement: Supplementary file 1 — Supplementary file1 (DOCX 45 KB) [file 10198_2025_1817_MOESM1_ESM.docx]

## Supplementary materials

**Table 4.** Cohort characteristics per clinic and per group

|  | **Standard model of care** | | **APP model of care** | |
| --- | --- | --- | --- | --- |
|  | **Horsens** | **Randers** | **Silkeborg** | **Viborg** |
| Cohort, n | 4014 | 3547 | 3384 | 2576 |
| Age, mean ± SD | 50.8 ± 15.7 | 51.1 ± 15.9 | 51.3 ± 15.1 | 50 ± 15.9 |
| Female, % | 50.2% | 47.9% | 51.5% | 51.1% |
| Civil status, n (%) |  |  |  |  |
| -Married^1^ | 2341 (58.3%) | 2008 (56.6%) | 2066 (61.1%) | 1540 (59.8%) |
| -Divorced or widowed^2^ | 735 (18.3%) | 691 (19.5%) | 617 (18.2%) | 441 (17.1%) |
| -Not married | 919 (22.9%) | 816 (23.0%) | 683 (20.2%) | 580 (22.5%) |
| -Unknown | 19 (0.5%) | 32 (0.9%) | 18 (0.5%) | 15 (0.6%) |
| Charlson Index, mean ± SD | 0.27 ± 0.72 | 0.29 ± 0.76 | 0.29 ± 0.74 | 0.29 ± 0.78 |
| Upper extremity diagnoses, n (%) |  |  |  |  |
| -Shoulder disorders^3^ | 2315 (57.7%) | 1575 (44.4%) | 2074 (61.3%) | 1484 (57.6%) |
| -Frozen shoulder | 187 (4.7%) | 264 (7.4%) | 362 (10.7%) | 142 (5.5%) |
| -Injury or sequela of UE^4^ | 87 (2.2%) | 66 (1.9%) | 45 (1.3%) | 79 (3.1%) |
| -Osteoarthritis^4^ | 775 (19.3%) | 1165 (32.8%) | 337 (10.0%) | 493 (19.1%) |
| -Luxation or subluxation^4^ | 639 (15.9%) | 466 (13.1%) | 550 (16.3%) | 362 (14.1%) |
| -Other | 11 (0.3%) | 11 (0.3%) | 16 (0.5%) | 16 (0.6%) |

APP: Advanced practice physiotherapy; SD: Standard deviation; UE: Upper extremity

1. Include registered partnership
2. Include dissolved partnership
3. Excluding frozen shoulder
4. Not joint-specific

**Table 5.** Care received per clinic and per group

|  | **Standard medical model of care** | | **APP model of care** | |
| --- | --- | --- | --- | --- |
|  | **Horsens** | **Randers** | **Silkeborg** | **Viborg** |
| Participants, n | 4014 | 3547 | 3384 | 2576 |
| Visits, mean ± SD |  |  |  |  |
| -Physician | 21.31 ± 19.59 | 20.93 ± 18 | 21.53 ± 19.1 | 21.6 ± 18.66 |
| -Physiotherapist | 3.9 ± 11 | 4.26 ± 11 | 3.8 ± 10.83 | 3.47 ± 10.8 |
| -Chiropractor | 0.52 ± 2.61 | 0.37 ± 2.18 | 0.38 ± 2 | 0.38 ± 2.74 |
| -Orthopedic surgeon | 0.03 ± 0.35 | 0.06 ± 0.57 | 0.04 ± 0.46 | 0.02 ± 0.21 |
| Hospital, mean ± SD |  |  |  |  |
| -Admission number | 0.28 ± 1.11 | 0.28 ± 1.1 | 0.29 ± 1.03 | 0.27 ± 1.26 |
| -Admission days | 0.76 ± 2.19 | 0.81 ± 2.06 | 0.78 ± 2.13 | 0.79 ± 2.02 |
| -Ambulatory visit | 2.03 ± 8.38 | 2.11 ± 9.88 | 2.1 ± 10.18 | 2.26 ± 13.28 |

APP: Advanced practice physiotherapy; SD: Standard deviation; UE: Upper extremity

**Table 6.** Mean healthcare cost and productivity loss per group and between-group differences per patients

|  | **Standard medical model of care n=7561** | **APP model of care n=5960** | **Between-group difference** |
| --- | --- | --- | --- |
|  | **Mean € (95% CI)** | **Mean € (95% CI)** | **Mean € (95% CI)^1^** |
| Primary care medical | 443 (432 to 453) | 445 (435 to 456) | 4 (-17 to 26) |
| Primary care rehabilitation | 93 (89 to 98) | 112 (107 to 118) | 16 (6 to 26) |
| Medications | 65 (60 to 70) | 15 (12 to 17) | -51 (-58 to -43) |
| Hospital total | 3215 (3034 to 3397) | 3309 (3098 to 3519) | 102 (-290 to 493) |
| *Hospital upper extremity^2^* | *1160 (1129 to 1191)* | *1090 (1059 to 1121)* | *-70 (-132 to -7)* |
| Total healthcare cost^3^ | 3817 (3632 to 4002) | 3881 (3667 to 4095) | 71 (-328 to 471) |
| Productivity loss | 15733 (14849 to 16617) | 16122 (15132 to 17112) | -125 (-952 to 701) |

APP: Advanced practice physiotherapy; CI: Confidence interval; MSK: Musculoskeletal

1. Positive between-group difference indicates higher costs per patients in the APP model of care.
2. Hospital upper extremity include cost related to episodes of care for an upper extremity disorders based on the ICD-10 codes
3. Total healthcare costs = Primary care medical + Primary care rehabilitation + Medications + Hospital total

**Table 7.** Propensity score adjusted mean healthcare cost and productivity loss per clinic per patients

|  | **Standard medical model of care** | | **APP model of care** | |
| --- | --- | --- | --- | --- |
|  | **Horsens; n=4013** | **Randers; n=3547** | **Silkeborg; n=3387** | **Viborg; n=2577** |
|  | **Mean € (95% CI)** | **Mean € (95% CI)** | **Mean € (95% CI)** | **Mean € (95% CI)** |
| Primary care medical | 444 (428 to 461) | 444 (431 to 457) | 444 (430 to 458) | 444 (428 to 460) |
| Primary care rehabilitation | 84 (78 to 90) | 105 (98 to 112) | 110 (103 to 118) | 114 (105 to 123) |
| Medications | 115 (105 to 125) | 8 (7 to 9) | 20 (16 to 23) | 8 (6 to 10) |
| Hospital total | 3229 (2933 to 3525) | 3208 (3012 to 3403) | 3226 (2998 to 3454) | 3407 (3029 to 3785) |
| *Hospital upper extremity^1^* | *1122 (1081 to 1163)* | *1202 (1155 to 1250)* | *1115 (1075 to 1156)* | *1057 (1008 to 1106)* |
| Total healthcare cost^2^ | 3873 (3571 to 4175) | 3764 (3564 to 3964) | 3800 (3566 to 4035) | 3973 (3590 to 4356) |
| Productivity loss | 15737 (14522 to 16952) | 15900 (14601 to 17200) | 17035 (15673 to 18397) | 14666 (13248 to 16085) |

APP: Advanced practice physiotherapy; CI: Confidence interval; MSK: Musculoskeletal

1. Hospital upper extremity include cost related to episodes of care for an upper extremity disorders based on the ICD-10 codes
2. Total healthcare costs = Primary care medical + Primary care rehabilitation + Medications + Hospital total

**Table 8.** Mean healthcare cost and productivity loss per clinic per patients

|  | **Standard medical model of care** | | **APP model of care** | |
| --- | --- | --- | --- | --- |
|  | **Horsens; n=4014** | **Randers; n=3547** | **Silkeborg; n=3384** | **Viborg; n=2576** |
|  | **Mean € (95% CI)** | **Mean € (95% CI)** | **Mean € (95% CI)** | **Mean € (95% CI)** |
| Primary care medical | 443 (427 to 460) | 442 (429 to 455) | 445 (431 to 459) | 446 (431 to 462) |
| Primary care rehabilitation | 84 (78 to 90) | 104 (97 to 111) | 111 (104 to 118) | 114 (105 to 123) |
| Medications | 115 (105 to 126) | 8 (7 to 9) | 20 (16 to 24) | 8 (6 to 10) |
| Hospital total | 3228 (2934 to 3523) | 3201 (3005 to 3397) | 3220 (2993 to 3447) | 3425 (3041 to 3810) |
| *Hospital upper extremity^1^* | *1123 (1082 to 1165)* | *1202 (1154 to 1249)* | *1118 (1077 to 1158)* | *1053 (1004 to 1102)* |
| Total healthcare cost^2^ | 3871 (3571 to 4171) | 3755 (3554 to 3955) | 3795 (3562 to 4029) | 3994 (3604 to 4383) |
| Productivity loss | 15622 (14414 to 16830) | 15859 (14561 to 17158) | 17194 (15827 to 18562) | 14714 (13294 to 16134) |

Advanced practice physiotherapy; CI: Confidence interval; MSK: Musculoskeletal

1. Hospital upper extremity include cost related to episodes of care for an upper extremity disorders based on the ICD-10 codes
2. Total healthcare costs = Primary care medical + Primary care rehabilitation + Medications + Hospital total
